# Supplementary material for: Total Burden of Cerebral Small Vessel Disease on MRI May Predict Cognitive Impairment in Parkinson’s Disease
Source: J Clin Med. 2022 Sep 14;11(18):5381. doi: 10.3390/jcm11185381 (PMC9501874; doi:10.3390/jcm11185381)
Supplement: Supplementary file 1 [file jcm-11-05381-s001.zip › jcm-1862480-supplementary.pdf]

Table S1. Multivariate binary logistic regression analyses of clinical factors associated with cognitive impairment in PD.

	OR	95%CI	*p*
Education level	0.285	0.171–0.476	0.000 *
PVH	2.523	1.232–5.167	0.011 *
CSVD	2.583	1.342–4.969	0.004 *

Note: * $p < 0.05$.

Table S2. Correlation analysis of Education level and clinical factors

	**r**	***p***
Midbrain-EPVS	0.010	0.915
SLI	−0.159	0.081
CMB	−0.042	0.648
DWMH	0.049	0.592
PVH	0.009	0.919
CS-EPVS	0.046	0.681
BG-EPVS	−0.051	0.579
CSVD	−0.153	0.093
